# Supplementary figures and images for: Anticariogenic Activity of Celastrol and Its Enhancement of Streptococcal Antagonism in Multispecies Biofilm
Source: Antibiotics (Basel). 2023 Jul 28;12(8):1245. doi: 10.3390/antibiotics12081245 (PMC10451999; doi:10.3390/antibiotics12081245)

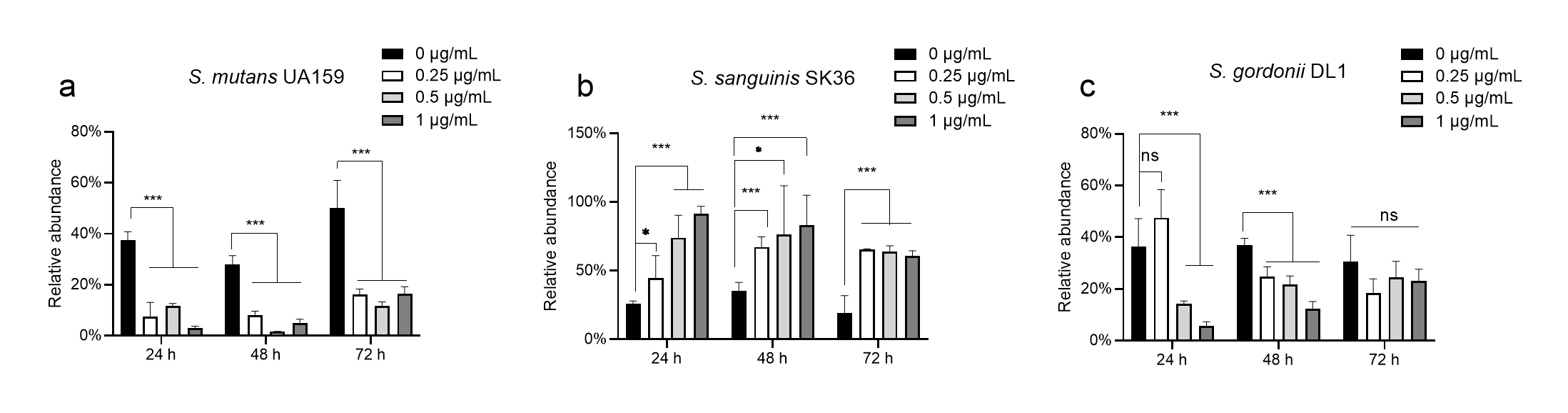

Supplement: Supplementary file 1 [file antibiotics-12-01245-s001.zip › Figure S1.tif]

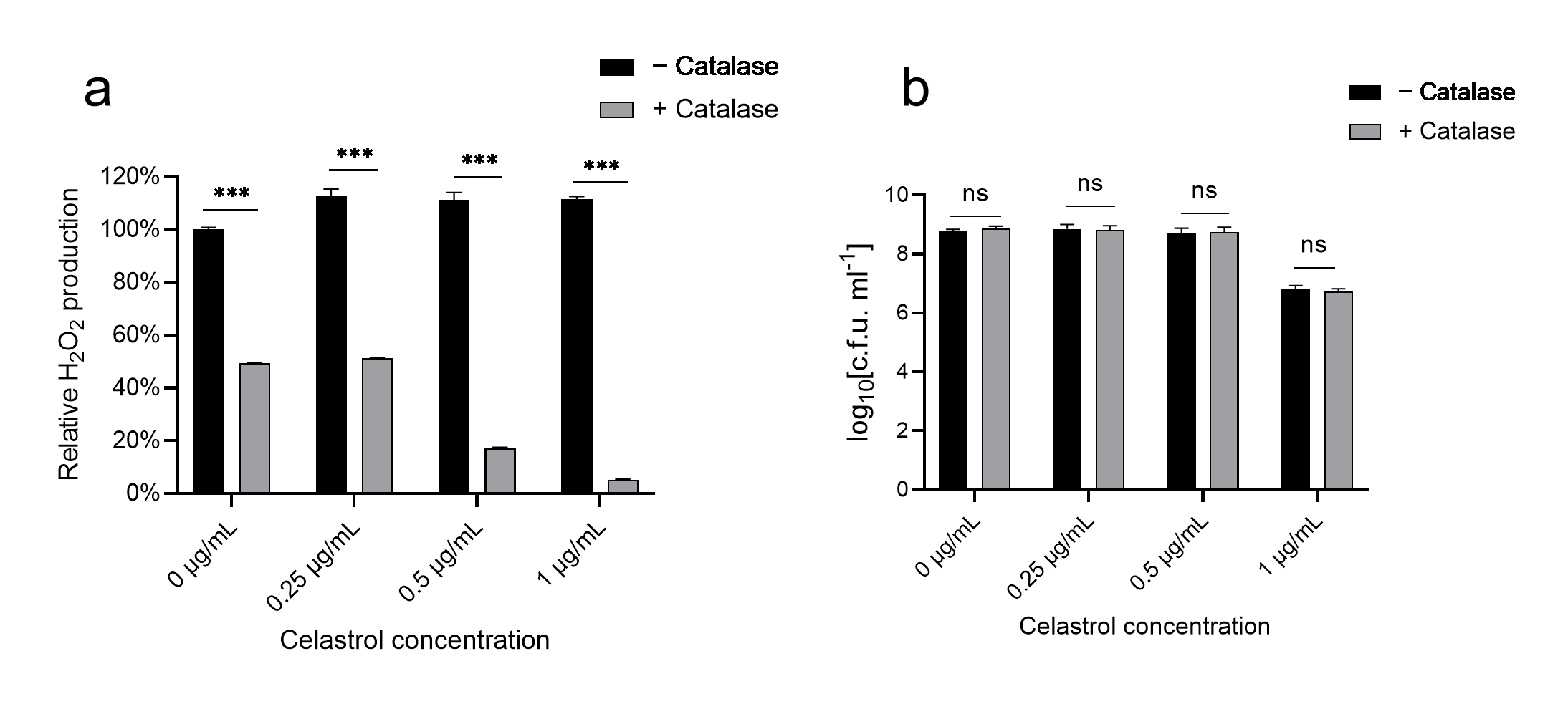

Supplement: Supplementary file 1 [file antibiotics-12-01245-s001.zip › Figure S2.tif]

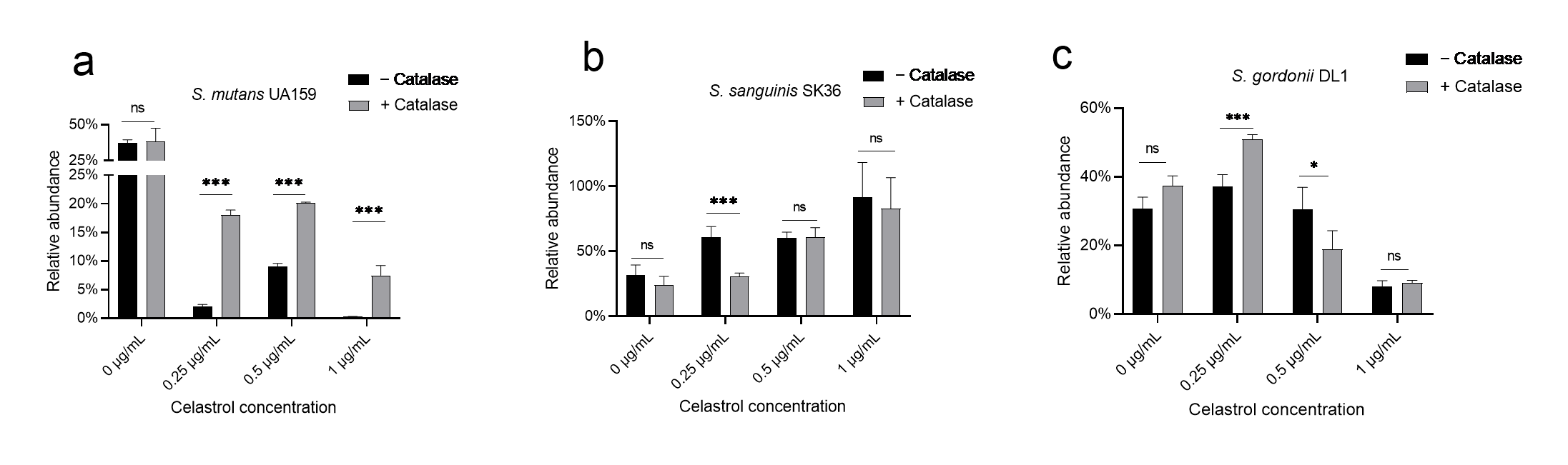

Supplement: Supplementary file 1 [file antibiotics-12-01245-s001.zip › Figure S3.tif]

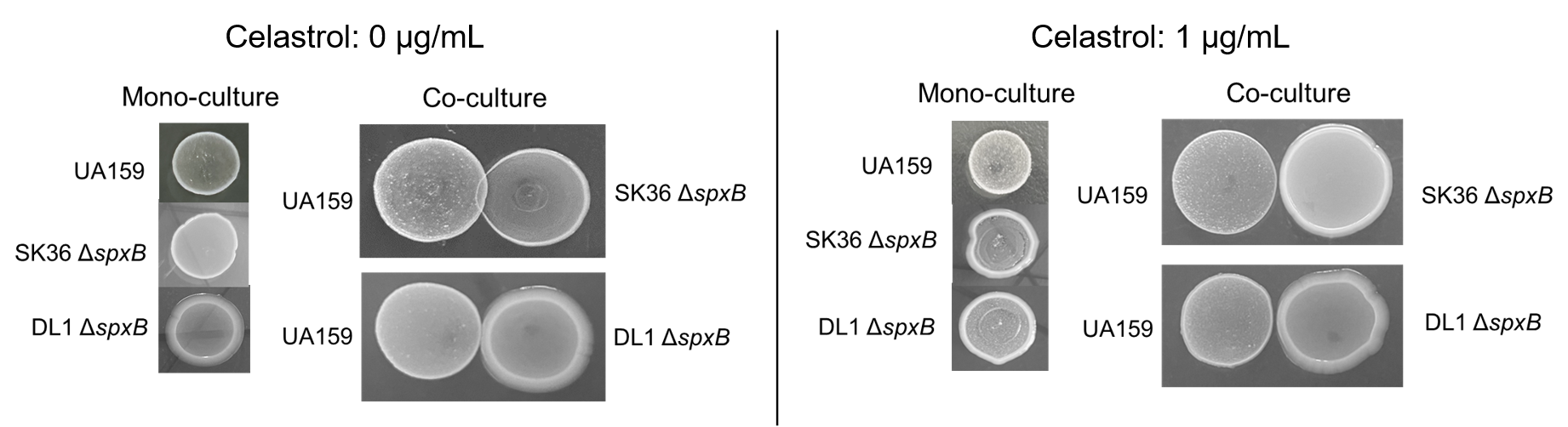

Supplement: Supplementary file 1 [file antibiotics-12-01245-s001.zip › Figure S4.tif]
